# Energy expenditure, intake and availability in female soccer players via doubly labelled water: Are we misrepresenting low energy availability?

**DOI:** 10.1113/EP091589

**Published:** 2024-08-15

**Authors:** Samuel J. McHaffie, Carl Langan‐Evans, Juliette A. Strauss, José L. Areta, Christopher Rosimus, Martin Evans, Ruth Waghorn, James Grant, Matthew Cuthbert, Catherine Hambly, John R. Speakman, James P. Morton

**Affiliations:** ^1^ Research Institute for Sport and Exercise Sciences (RISES) Liverpool John Moores University Liverpool UK; ^2^ The Football Association, Needwood Burton‐Upon‐Trent UK; ^3^ Institute of Biological and Environmental Sciences University of Aberdeen Aberdeen UK

**Keywords:** association football, carbohydrate, football, REDs

## Abstract

Female soccer players have been identified as presenting with low energy availability (LEA), though the prevalence of LEA may be overestimated given inaccuracies associated with self‐reporting dietary intakes. Accordingly, we aimed to quantify total daily energy expenditure (TDEE) via the doubly labelled water (DLW) method, energy intake (EI) and energy availability (EA). Adolescent female soccer players (*n* = 45; 16 ± 1 years) completed a 9–10 day ‘training camp’ representing their national team. Absolute and relative TDEE was 2683 ± 324 and 60 ± 7 kcal kg^−1^ fat free mass (FFM), respectively. Mean daily EI was lower (*P* < 0.01) when players self‐reported using the remote food photography method (RFPM) (2047 ± 383 kcal day^−1^) over a 3‐day period versus DLW derived EI estimates accounting for body mass (BM) changes (2545 ± 518 kcal day^−1^) over 7–8 days, representing a mean daily Δ of 499 ± 526 kcal day^−1^ and 22% error when using the RFPM. Estimated EA was different (*P* < 0.01) between methods (DLW: 48 ± 14 kcal kg^−1^ FFM, range: 22–82; RFPM: 37 ± 8 kcal kg^−1^ FFM, range: 22–54), such that prevalence of LEA (<30 kcal kg^−1^ FFM) was lower in DLW compared with RFPM (5% vs. 15%, respectively). Data demonstrate the potential to significantly underestimate EI when using self‐report methods. This approach can therefore cause a misrepresentation and an over‐prevalence of LEA, which is the underlying aetiology of ‘relative energy deficiency in sport’ (REDs).

## Abstract (highlights)

- **What is the central question of this study** **?**
  Do self‐reported dietary intakes (via remote food photography method, RFPM) overestimate low energy availability (LEA) prevalence in female soccer players compared with energy intake evaluation from the doubly labelled water (DLW) method?
- **What is the main finding and its importance?**
  Estimated energy availability is greater with the DLW method compared with RFPM, such that the prevalence of LEA is greater when self‐reporting dietary intakes. Accordingly, data demonstrate the potential to misrepresent the prevalence of LEA, an underlying factor in the aetiology of ‘relative energy deficiency in sport’ (REDs).

## 1 INTRODUCTION

In 2023, the International Olympic Committee published their most recent consensus statement on ‘relative energy deficiency in sport’ (REDs), defined as ‘a syndrome of impaired physiological and psychological functioning caused by exposure to problematic (prolonged and/or severe) low energy availability (LEA)’ (Mountjoy et al., 2023a). Although it is suggested that REDs may occur in both female and male athletes (Ackerman et al., 2019), the study of LEA (which is the underlying aetiology of REDs) is more prominent in female athletes as a result of the historical assumption that this syndrome exclusively impacted female athletes (Nattiv & Lynch, 1994). However, in recognising that LEA may compromise other physiological symptoms beyond that of bone health and menstrual function (De Souza et al., 2019; Nattiv et al., 2021), it is now acknowledged within the latest REDs consensus statement that ‘REDs may present as decreased energy metabolism, reproductive function, musculoskeletal health, immunity, glycogen synthesis and cardiovascular health, the result of which is associated with impaired well‐being, increased injury risk and decreased sports performance in both male and female athletes’ (Mountjoy et al., 2023a).

In considering that LEA is an exposure variable at the centre of the REDs health and performance conceptual models (that when problematic may result in various deleterious symptoms outlined in the REDs model), it is unsurprising that REDs is most frequently studied in the context of those athletes that routinely present with high daily energy expenditure and/or sub‐optimal energy intake (e.g., gravitational, weight‐categorised and aesthetic sports). In addition to the aforementioned sports, a growing body of literature also suggests that LEA is evident in female soccer players (Dasa et al., 2023; McHaffie et al., 2023; Morehen et al., 2021; Moss et al., 2021; Reed et al., 2014). For example, in categorising LEA using traditional laboratory derived classifications of <30 kcal kg^−1^ fat free mass (FFM) day^−1^ (Loucks & Thuma, 2003; Loucks et al., 1998), we have recently reported a high prevalence of LEA in both international standard adult (Morehen et al., 2021) and adolescent (McHaffie et al., 2023) female soccer players of 88% and 34%, respectively. It is acknowledged, however, that the adoption of a cut‐off point of <30 kcal kg^−1^ FFM day^−1^ is grounded in short‐term laboratory studies (Ihle & Loucks, 2004; Loucks et al., 1998; Loucks & Thuma, 2003), with recent real‐life clinical investigations challenging the appropriateness of a singular, universal threshold in free‐living athletes (Burke et al., 2018; Deutz et al., 2000; Fahrenholtz et al., 2018). Indeed, recent research reveals considerable variations in the energy availability (EA) values that are linked to adverse health and performance outcomes across individuals, sex and diverse bodily systems (De Souza et al., 2022; Lieberman et al., 2018; Salamunes et al., 2024).

In considering that EA is calculated as the difference between dietary energy intake (EI) and exercise related energy expenditure (EEE) expressed relative to the individual's FFM (Loucks et al., 2011), the cause of LEA in female soccer players has been largely attributed to sub‐optimal EI (Dasa et al., 2023; Morehen et al., 2021; Moss et al., 2021). This assertion is based on the observation that the habitual training volume and associated total daily energy expenditure (TDEE) of such players is relatively comparable to male soccer players (Anderson et al., 2017) and moreover, is not considered excessive when compared with female endurance athletes (Heydenreich et al., 2017; Schulz et al., 1992). Furthermore, in using a qualitative research methodology, we also reported a culture of ‘carbohydrate fear’ amongst female soccer players, where such athletes may intentionally practice periods of deliberate ‘under‐fuelling’ due to a fear of gaining body fat and perceived external pressure from staff members and social media (McHaffie et al., 2022). Notwithstanding these recent data, it is also important to adopt a more critical lens when evaluating the true extent of LEA in female soccer players (Parker et al., 2022). Indeed, it is well documented that assessment of dietary intake has known limitations including conscious or unconscious under‐reporting (Gemming et al., 2014; Livingstone & Black, 2003; Martin et al., 2012; Rollo et al., 2016; Thompson et al., 2010) alongside both intra‐ and inter‐coder error when evaluating dietary records (Stables et al., 2021), especially in the context of the recently popularised remote food photographic method (RFPM). Accordingly, when we applied a recently published correction factor (22%) to account for potential dietary under‐reporting in female soccer players (Dasa et al., 2023), we observed that the prevalence of LEA (albeit defined as <30 kcal kg^−1^ FFM day^−1^) was reduced from 34% to 5% in international female soccer players (McHaffie et al., 2023).

To overcome such methodological limitations, an alternative method for measuring EI is to utilise the doubly labelled water (DLW) technique (as the gold standard method to directly assess TDEE in free living conditions) alongside measuring changes in body mass (BM) over a known duration (de Jonge et al., 2007; Pieper et al., 2011; Tarnowski et al., 2023). In this way, the DLW method can be used to calculate EI and reduce the error associated with self‐reporting dietary intake (Capling et al., 2017; Poslusna et al., 2009). Although we also acknowledge that inaccuracies exist beyond EI when measuring EA (e.g., surrounding the definition of ‘exercise’, assessment of EEE and measurement of FFM; Areta et al., 2021), the use of DLW‐derived estimates of EI is still likely to lead to a more accurate evaluation of the true prevalence of LEA in this population. This consideration gains added significance since research employing DLW has been conducted to a lesser extent in female athletes compared to male athletes. This disparity is noteworthy especially since female athletes seem to be more adversely affected by LEA, in comparison to male athletes (Areta et al., 2021; Koehler et al., 2016).

With this in mind, the aim of the present study was to utilise the DLW method to quantify TDEE, EI and EA in female soccer players. In using the largest total sample size studied to date (*n* = 45), we assessed a cohort of international adolescent female soccer players during which they took part in an extended training camp where they completed a training and game schedule representing their national team. We specifically hypothesised that EA would be significantly greater when calculated from DLW‐derived assessments of EI versus those estimates of EI derived from the RFPM.

## 2 METHODS

### 2.1 Ethics approval

The study was conducted according to the *Declaration of Helsinki* and was approved by the University Ethics Committee of Liverpool John Moores University (22/SPS/027). All experimental procedures and associated risks were explained to players and written informed consent provided, with parental/guardian consent and player assent also obtained for participants <18 years old.

### 2.2 Participants

Forty‐five female international soccer players competing at international under 17 (U17s) (*n *= 17) and under 19 (U19s) (*n *= 28) level volunteered to take part in the study. Participant characteristics categorised by squad and as a whole sample cohort are presented in Table 1.

**TABLE 1:** Participant characteristics.

	Under 17s (*n* = 17)	Under 19s (*n* = 28)	Whole sample (*n* = 45)
Age (years)	16 ± 1	18 ± 1	17 ± 1
Stature (cm)	168.4 ± 6.1	168.0 ± 5.9	168.1 ± 6.0
Body mass (kg)	60.3 ± 8.4	61.1 ± 6.0	60.8 ± 7.0
Fat free mass (kg)	46.1 ± 5.2	44.3 ± 4.9	45.0 ± 5.1
Fat mass (kg)	14.9 ± 5.0	16.8 ± 3.7	16.1 ± 4.3
Body fat (%)	23.9 ± 6.1	27.3 ± 4.4	26.1 ± 5.4
Predicted RMR (kcal day^−1^)	1457 ± 92	1457 ± 70	1457 ± 79

*Note*: Mean age, stature, BM, FFM, fat mass and percent body fat values are presented for each squad and as a whole sample. Predicted RMR was calculated according to Roza and Shizgal (1984).

### 2.3 Overview of study design

Data were collected from participants during an international training and match fixture camp, lasting 10 days (U17s) and 9 days (U19s), respectively. The U17s camp featured three match days, four training days and three rest days, whilst the U19s camp comprised two match days, five training days and two rest days, as outlined in Tables 2 and 3. Tables 2 and 3 also detail the external load of each camp, presented as daily values coded relative to the match days (e.g., MD−1 is the day before the match) and total values. The DLW technique was used to measure TDEE throughout the data collection period. During each camp, all the players self‐reported their dietary intake using the RFPM on MD−1, MD and MD+1, and EI was also calculated based on the DLW derived assessment of TDEE, in combination with changes in BM (Tarnowski et al., 2023). Pitch‐based training and match load were measured using global positioning system (GPS) technology. EEE was estimated using GPS technology and ratings of perceived exertion (RPE) for on‐pitch and gym‐based strength and conditioning sessions, respectively. FFM data were derived using the DLW technique and in combination with EI (using both methods) and EEE data was used to calculate EA.

**TABLE 2:** An overview of external loading throughout the 10‐day period of the under 17s training and fixture period.

	MD−2	MD−1	MD	MD+1	MD−1	MD	MD+1	MD−1	MD	MD+1	Mean total
Duration (min)	76 ± 3	70 ± 0	69 ± 40	0	73 ± 4	79 ± 32	0	62 ± 0	82 ± 0 41	0	512 ± 77
Distance (m)	6184 ± 457	4721 ± 386	6326 ± 4014	0	4308 ± 435	6995 ± 3031	0	3932 ± 318	7650 ± 4740	0	40,116 ± 8786
Distance covered at speed zone 1 (3.46–5.28 m s^−1^) (m)	930 ± 218	691 ± 81	998 ± 746	0	699 ± 129	1025 ± 520	0	594 ± 139	1301 ± 1030	0	6238 ± 2001
Distance covered at speed zone 2 (5.29–6.25 m s^−1^) (m)	358 ± 263	213 ± 169	229 ± 276	0	296 ± 214	234 ± 204	0	193 ± 133	368 ± 403	0	1890 ± 1416
Distance covered at speed zone 3 (≥6.26 m s^−1^) (m)	74 ± 47	22 ± 13	66 ± 50	0	48 ± 45	88 ± 68	0	34 ± 29	157 ± 126	0	488 ± 257
Frequency of accelerations (>3 m s^−1^) (AU)	40 ± 10	43 ± 10	34 ± 0 23	0	44 ± 11	38 ± 16	0	38 ± 10	44 ± 25	0	281 ± 69
Frequency of decelerations (>3 m s^−1^) (AU)	48 ± 13	50 ± 11	39 ± 30	0	45 ± 9	48 ± 23	0	39 ± 9	53 ± 35	0	321 ± 89

*Note*: Daily and overall pitch‐based training and match duration (min), distance (m), distance at speed zone 1 (m), distance at speed zone 2 (m), distance at speed zone 3 (m), frequency of accelerations (AU) and frequency of decelerations (AU).

**TABLE 3:** An overview of external loading throughout the 9‐day period of the under 19s training and fixture period.

	MD−2	MD−1	MD	MD+1	MD−3	MD−2	MD−1	MD	MD+1	Mean total
Duration (min)	88 ± 26	79 ± 6	120 ± 30	0	106 ± 24	113 ± 19	83 ± 35	92 ± 59	0	680 ± 91
Distance (m)	4197 ± 1656	4092 ± 1417	8401 ± 309	0	5320 ± 1307	4875 ± 1207	3994 ± 1851	5129 ± 4064	0	36,009 ± 5836
Distance covered at speed zone 1 (3.46–5.28 m s^−1^) (m)	509 ± 274	445 ± 288	1328 ± 691	0	602 ± 201	559 ± 283	430 ± 324	897 ± 862	0	4770 ± 1573
Distance covered at speed zone 2 (5.29–6.25 m s^−1^) (m)	111 ± 134	119 ± 164	273 ± 178	0	135 ± 117	171 ± 134	132 ± 190	165 ± 139	0	1106 ± 632
Distance covered at speed zone 3 (≥6.26 m s^−1^) (m)	48 ± 86	35 ± 43	126 ± 182	0	78 ± 146	79 ± 150	80 ± 165	62 ± 80	0	509 ± 651
Frequency of accelerations (>3 m s^−1^) (AU)	29 ± 12	45 ± 17	56 ± 18	0	40 ± 15	33 ± 11	41 ± 20	31 ± 27	0	276 ± 60
Frequency of decelerations (>3 m s^−1^) (AU)	35 ± 18	43 ± 16	64 ± 24	0	49 ± 20	34 ± 13	39 ± 20	37 ± 35	0	301 ± 84

*Note*: Daily and overall pitch‐based training and match duration (min), distance (m), distance at speed zone 1 (m), distance at speed zone 2 (m), distance at speed zone 3 (m), frequency of accelerations (AU) and frequency of decelerations (AU).

### 2.4 Quantification of external training and match load

Training and match load were measured using GPS technology (Apex, STATSports, Newry, UK), with units worn by all outfield players (*n* = 40) for all the pitch‐based training sessions and matches. The GPS units were placed inside custom‐made manufacturer‐provided vests (Apex, STATSports), which were positioned on the players’ upper‐back between both scapulae, allowing the exposure of the GPS antennae to acquire a clear satellite connection. Variables measured included session duration in minutes (min), distance covered in metres (m), frequency of accelerations (an increase in speed of >3 m s^−1^), frequency of decelerations (a decrease in speed of <3 m s^−1^) and time spent in three different speed zones: zone 1 (3.46−5.28 m s^−1^), zone 2 (5.29−6.25 m s^−1^) and zone 3 (≥6.26 m s^−1^). These categories are commonly used within this population, as established by Park et al. (2019).

### 2.5 During exercise energy expenditure

For pitch‐based sessions, GPS devices with individualised player descriptives inputted provided gross EEE values, which were subsequently increased by 10.7%, based on recent data demonstrating that this GPS system underestimates EEE within this population (Dasa et al., 2022). Gross EEE values for pitch‐based sessions were converted into net EEE using resting metabolic rate (RMR) estimations based on the updated Harris–Benedict equation (Roza & Shizgal, 1984), whereby the energy expended for RMR during the session and thermic effect of food (assumed to be 10%) were subtracted from the estimation of gross EEE, resulting in a value of net EEE. For gym‐based sessions, EEE was estimated based on the timing of each individual session, the participant's RPE and the content of each session. Using these data, each gym session could be converted into a metabolic‐equivalent task (MET) for the assigned period (Butte et al., 2018), allowing an estimation of EEE for each individual session. Physical activity level (PAL) was also determined by dividing the TDEE by RMR.

### 2.6 Measuring of energy expenditure using the DLW technique

TDEE (kJday^−1^) was estimated using the DLW technique (Lifson & McClintock, 1966). This method has been previously validated on multiple occasions by comparison to simultaneous indirect calorimetry in humans (Speakman, 1997). A pre‐dosing urine sample was collected during the evening of day zero, to estimate background isotope enrichments for each individual. Participants were weighed and then dosed orally by drinking a weighed quantity of mixed isotopes provided by the University of Aberdeen in a sealed bottle. This single bolus oral dose was weighed to four decimal places of deuterium (^2^H_2_) and oxygen (^18^O) stable isotopes in the form of water (^2^H_2_^18^O), with a desired enrichment of 10% ^18^O and 5% ^2^H_2_ using the calculation:

$$\mathrm{Dose}\;\left(\mathrm{mL}\right)=0.65\left(\mathrm{BM},g\right)\;\times \mathrm{DIE}/\mathrm{IE},$$

where 0.65 is the approximate proportion of the body comprised of water, DIE is the desired initial enrichment (DIE = 618.923 BM (kg) − 0.305) and IE is the initial enrichment (100,000 parts per million) (Speakman, 1997), dosed according to BM two weeks prior to the national training camp. To ensure the whole dose was administered, participants were observed consuming each bolus, and each glass vial was refilled with additional water, which players were asked to consume. Time of dosing and urine sample were recorded throughout the study period. Urine samples were collected on the morning of day 1, approximately 12 h post‐dose, to obtain the initial isotope enrichments, following total body water equilibrium (Speakman, 1997). Further urine samples (2nd void of the day) were collected daily until the last day of the training camp. In addition, daily samples were also collected from a non‐dosed individual of the team, to adjust for variations in the background level of the isotopes, which may have occurred when travelling to a new country during the early stages of the study. All urine samples were collected by the participants in a 100 mL urine pot, before being immediately transferred into 1.5 mL cryovials. All samples were then kept frozen (−17.8°C) and then transported on dry ice, until subsequent analysis. Analysis of the isotopic enrichment of urine was performed blind, using a Liquid Isotope Water Analyser (DLT‐100, Los Gatos Research, California, USA) (Berman et al., 2013). Initially the urine was vacuum distilled (Nagy et al., 1993), and the resulting distillate was used for analysis. Samples were run alongside five lab standards for each isotope and international standards to adjust for daily machine variation and correct delta values to parts per million. Daily isotope enrichments were log_e_ converted after adjusting for the background fluctuations, and the elimination constants (*K*_o_ and *K*_d_) were calculated by fitting a least squares regression model to the log_e_‐converted data. The back extrapolated intercept was used to calculate the isotope dilution spaces (*N*_o_ and *N*_d_). A two‐pool model, specifically Speakman (1997), was used to calculate rates of CO_2_ production.

### 2.7 Assessment of energy intake using the remote food photography method

Self‐reported energy and macronutrient intake was quantified on three days. These days corresponded with MD−1, MD and MD+1 for the U17s (days 5, 6 and 7) and U19s (days 2, 3 and 4), measured using the RFPM (Martin et al., 2012). Although this methodology has been questioned with regards to its accuracy when assessing EI (Capling et al., 2017), these data were collected for comparison to the DLW assessment of EI and to provide insights into macronutrient intake. As the lead researcher was present for the entire training camp, reminders were made in person throughout the entire data collection period, alongside physical prompts being placed in the dining area to further enhance compliance. As per the protocol, participants were instructed to take two images, at 90° and 45° of any food or drink they consumed throughout the three days, including all meals and snacks. A third image was also taken of any leftovers, if required. These images were sent to the lead researcher via the mobile telephone application Threema (Threema GmbH, Pfäffikon, Switzerland). Participants were also instructed to send a brief description of the food items that they consumed, including quantities, brands and food types. The lead researcher constructed two portions (small and large) of each of the foods available, prior to the arrival of players and staff members for meal times. These were weighed and photographed, providing detail to compare to during the analysis process, for a more accurate overview of portion size. Descriptions of foods and drinks consumed before, during and after training and matches were also recorded, as players did not have access to their phones at this time. Some players brought their own snacks to the training camp; however, all meals and multiple snacks were provided to players throughout each day, including during training matches. Once throughout the training camp, every player completed a dietary recall, to check for any missed data. Energy and macronutrient intake were analysed by a Sport and Exercise Nutrition register (SENr) accredited practitioner using the dietary analysis software Nutritics (Nutritics, v5, Dublin, Ireland), with energy and carbohydrate (CHO) intake quantified as kilocalories (kcal) and grams (g), respectively, in both absolute and relative (to each player's BM) terms. To ensure reliability of energy and macronutrient intake data, a second SENr nutritionist also analysed a sample of food diaries chosen at random (*n* = 5, equating to 15 days of entries in total), with interrater reliability determined via an independent Student's *t*‐test. No significant differences were observed between estimations for energy (*P* = 0.96; 95% CI: −156 to 38) and CHO (*P* = 0.11; 95% CI: −31 to 3) intake.

### 2.8 Assessment of energy intake using the doubly labelled water method

EI was calculated using the DLW method through the adjustment of TDEE for changes in energy stores, as outlined previously by Schulz et al. (1992). This was measured from day 1, up until the MD−1 before the last MD of camp 1 (day 8) and camp 2 (day 7). This time period was selected given BM is influenced to a greater extent by fluctuating muscle glycogen levels and total body water after an MD−1 and MD, due to CHO loading, in‐game nutritional practices, as well as the demands of match play (Bergström et al., 1967). BM (SECA, model 875, Class III, Hamburg, Germany) was collected daily when players were in a fasted state prior to breakfast on all days, between 08.00 and 08.45 h, immediately following the participants’ first urine pass of the day. Maintaining consistency in the timing of BM data collection was crucial for minimizing potential errors associated with daily fluctuations in BM levels, thereby reducing measurement variability. Players wore the same training kit and removed their shoes and any jewellery. The equation used was: EI (kcal day^−1^) = TDEE (kcal day^−1^) + change in energy stores (kcal in grams of body fat + kcal in grams of FFM change). As outlined by Schulz et al. (1992), this was estimated with the assumption that two‐thirds of change in BM was metabolic and one‐third was water, and that three‐quarters of the change in metabolic mass was fat mass and one‐quarter was FFM. For participants who lost BM, it was assumed 9 kcal g^−1^ of fat mass and 1 kcal g^−1^ of FFM and for BM gain, the assumption was 13.2 kcal g^−1^ of fat mass and 2.2 kcal g^−1^ of FFM (Forbes et al., 1986; Pullar & Webster, 1977; Spady et al., 1976).

### 2.9 Assessment of energy availability

EA for all outfield players was calculated using the formula EA = (EI − EEE)/FFM (Loucks et al., 1998), with FFM values established from the DLW method. This calculation was performed twice, first using EI data obtained via the DLW method, and second by employing the RFPM, to facilitate a comparison between these methodologies in measuring EA. Despite ongoing debates about the appropriateness of universal EA thresholds (Burke et al., 2018), this study classified players into categories of optimal EA (> 45 kcal kg^−1^ FFM  day^−1^), reduced EA (30–45 kcal kg^−1^ FFM day^−1^) and low EA (< 30 kcal kg^−1^ FFM day^−1^) to allow for comparative analysis with previous research on female soccer players (Dasa et al., 2023; McHaffie et al., 2023; Morehen et al., 2021; Moss et al., 2021). This categorization was employed alongside the presentation of raw EA values calculated by both methods, to provide a comprehensive assessment of energy status.

### 2.10 Statistical analysis

All data were initially assessed for normality of distribution via a visual inspection of histograms and a Shapiro–Wilk test. To determine differences between days in absolute and relative energy and macronutrient intake, EA and positional differences in TDEE, a univariate one‐way between groups analysis of variance (ANOVA) was used. Where significant main effects were present, Fisher's least significant difference *post hoc* analysis was conducted for pairwise comparisons. Ninety‐five percent confidence intervals (95% CI) for the differences are also presented. External training load, TDEE, TDEE relative to BM and TDEE relative to FFM were compared between U17 and U19 players using an independent Student's *t*‐test and changes in BM within squads were analysed using a paired sample *t*‐test. The strength of association between DLW and RFPM EI measurements was assessed using Pearson (*r*) correlation analysis, employing the following criteria to explain the relationship of association: trivial <0.1, small 0.1–0.29, moderate 0.3–0.49, large 0.5–0.69, very large 0.7–0.89 and almost perfect 0.9–1.00 (Hopkins et al., 2009). To assess the validity of the remote food photography method (RFPM), we evaluated its accuracy by calculating the percentage error in energy intake measurements taken with RFPM, using energy intake measured by the DLW method as the reference standard. The percentage error was determined by comparing the observed values from RFPM to the true values obtained from DLW. Additionally, the percentage difference between these two methods was calculated to further quantify discrepancies in measurement accuracy. Least squares regression was also used to assess validity, where DLW EI was regressed against RFPM EI measurements (Hopkins et al., 2009). Fixed bias was assessed by determining whether the intercept for the regression was different from zero and proportional bias was deemed present if the slope of the regression line was different from one. Random error was quantified using standard error of the estimate (SEE) from the regression. Predictive accuracy of each equation for individuals was calculated and evaluated based on the mean of 95% prediction interval (95% PI) for each regression equation. However, it is important to note that the literature currently lacks a universally accepted error rate for EI measurements, which limits the context for direct comparison of our findings. Relationships between daily EE and BM, FFM, stature, age, total distance and total duration, as well as energy balance (EB) using both methods of measuring EI were also assessed using Pearson's correlation. All statistical analyses were completed using SPSS Statistics (version 26; IBM Corp., Armonk, NY, USA) where *P* < 0.05 is indicative of statistical significance. All data are presented as means ± SD.

## 3 RESULTS

### 3.1 External loading patterns during both training camps

For illustrative purposes, an overview of outfield players’ external loading patterns for those players partaking in the U17s training camp (*n* = 15) and U19s training camp (*n* = 25) are presented in Tables 2 and 3, respectively. When comparing the 7 days of ‘exercise’ completed on both training camps, there was a significant difference in total duration of time ‘on pitch’ (*P* < 0.01) during the U19s camp (680 ± 93 min) compared with the U17s camp (512 ± 80 min; 95% CI: −227 to −110 min). However, there were no significant differences between camps for cumulative total distance (U17s: 40,116 ± 9095 m; U19s: 36,009 ± 5957 m; 95% CI : −701 to 8915 m; *P* = 0.09), total distance in speed zone 2 (U17s: 1890 ± 1466 m; U19s: 1106 ± 646 m; 95% CI: −58 to 1627 m; *P* = 0.07), distance in speed zone 3 (U17s: 488 ± 266 m; U19s: 509 ± 665 m; 95% CI: −385 to 345 m; *P* = 0.91), frequency of total accelerations (U17s: 281 ± 69; U19s: 276 ± 60; 95% CI: −37 to 47; *P* = 0.81) or frequency of total decelerations (U17s: 321 ± 92; U19s: 301 ± 86; 95% CI: −39 to 78 m; *P* = 0.5). In contrast, U17 players completed more accumulative distance (6238 ± 2071 min) in speed zone 1 versus U19 players (4770 ± 93 m; 95% CI: 284–2652 m; *P* = 0.02).

### 3.2 Total daily energy expenditure

**FIGURE 1:**
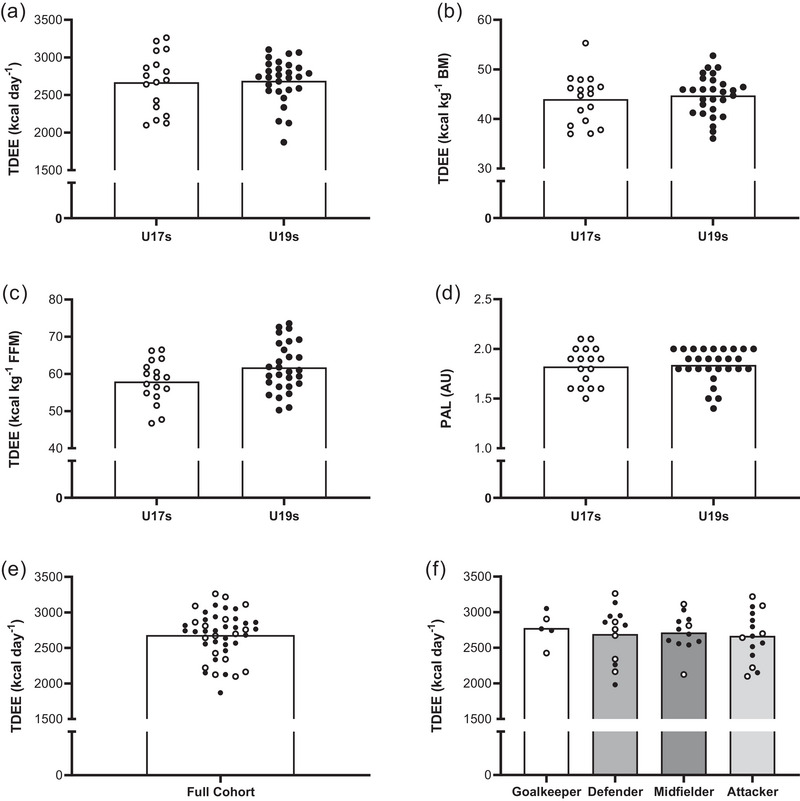
(a) Absolute TDEE, (b) TDEE relative to BM, (c) TDEE relative to FFM, (d) PAL, (e) TDEE of the entire cohort (*n* = 45), and (f) TDDE in each playing position. All individual circles represent an individual player, with open circles representing U17s (*n = *17) and filled circles representing U19s (*n = *28).

When comparing U17 and U19 players, there were no significant differences between mean absolute TDEE (U17s: 2671 ± 375 kcal day^−1^; U19s: 2689 ± 288 kcal day^−1^; 95% CI: −186 to 225 kcal day^−1^; *P* = 0.85; Figure 1a), TDEE relative to BM (U17s: 44 ± 5 kcal kg^−1^ BM day^−1^; U19s: 44 ± 4 kcal kg^−1^ BM day^−1^; 95% CI: −2 to 3.5 kcal kg^−1^ BM day^−1^; *P* = 0.60; Figure 1b), TDEE relative to FFM (U17s: 58 ± 6 kcal kg^−1^ FFM day^−1^; U19s: 61 ± 7 kcal kg^−1^ FFM day^−1^; 95% CI: −0.1 to 7.7 kcal kg^−1^ FFM day^−1^; *P* = 0.06; Figure 1c) and PAL (U17s: 1.8 ± 0.2; U19s: 1.8 ± 0.2; 95% CI: −0.1 to 0.1; *P* = 0.79; Figure 1d). Given that there was no difference in TDEE and PAL between squads, when all players were pooled, TDEE was 2683 ± 324 kcal day^−1^ (range: 1871–3262 kcal day^−1^; Figure 1e). Additionally, no significant differences were apparent when comparing TDEE between goalkeepers (2777 ± 232 kcal day^−1^), defenders (2651 ± 403 kcal day^−1^), midfielders (2707 ± 267 kcal day^−1^) and attackers (2659 ± 349 kcal day^−1^) (all *P* > 0.05) (Figure 1f).

### 3.3 Relationship of factors to TDEE

**FIGURE 2:**
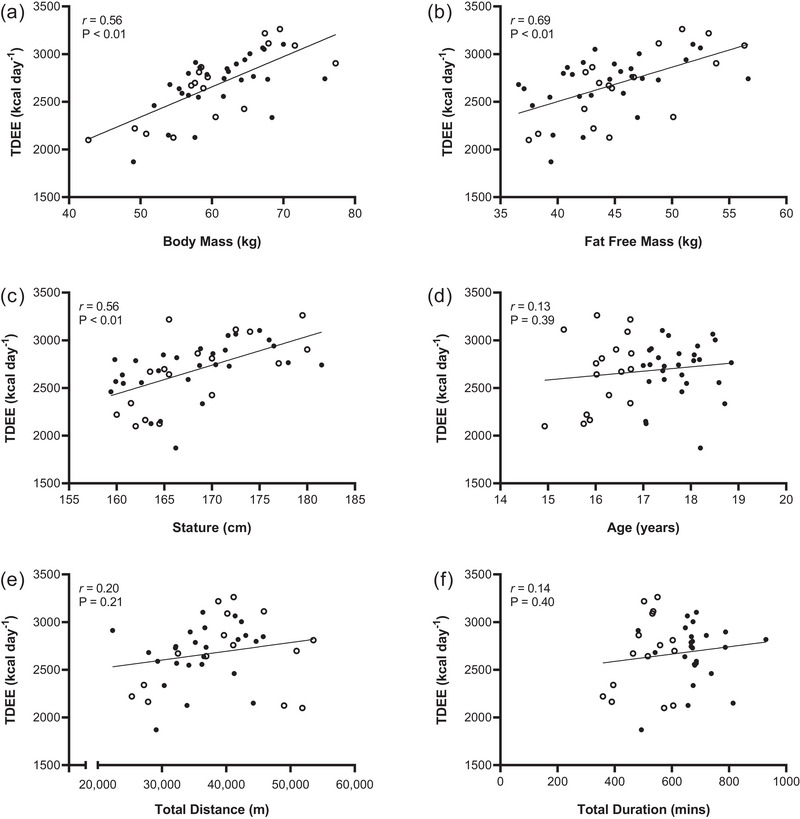
The relationships between TDEE and (a) BM, (b) FFM, (c) stature, (d) age, (e) total distance, and (f) total duration (*n* = 45 for a–d; *n* = 40 for e, f). All individual circles represent an individual player, with open circles representing U17s (*n* = 17) and filled circles representing U19s (*n* = 28).

There was a significant large positive relationship between TDEE and BM (*r* = 0.56; 95% CI: 0.37–0.72; *P* < 0.01) (Figure 2a), FFM (*r* = 0.69; 95% CI: 0.52–0.82; *P* < 0.01) (Figure 2b) and stature (*r* = 0.56; 95% CI: 0.32–0.73; *P* < 0.01) (Figure 2c). However, there was no significant relationship between TDEE and age (*r* = 0.13; 95% CI: −0.17 to 0.41; *P* = 0.39) (Figure 2d), total distance (*r* = 0.20; 95% CI: −0.12 to 0.48; *P* = 0.21) (Figure 2e) or total duration (*r* = 0.14; 95% CI: −0.18 to 0.3; *P* = 0.40) (Figure 2f).

### 3.4 Self‐reported energy and CHO intake via the RFPM

**FIGURE 3:**
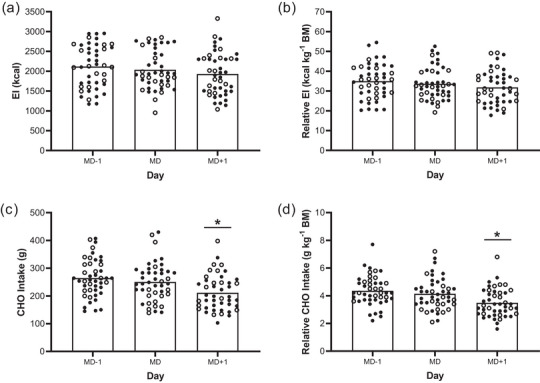
Absolute (a) and relative (b) EI and absolute (c), and relative (d) CHO intake across the 3‐day assessment period (*n* = 45 for all variables). All individual circles represent an individual player, with open circles representing U17s (*n = *17) and filled circles representing U19s (*n = *28). *Significant difference between days (*P <* 0.05).

Mean daily EI (*n* = 45) using the RFPM was 2047 ± 383 kcal day^−1^. There was no significant difference in absolute EI between MD−1 (2126 ± 531 kcal day^−1^) and MD (2073 ± 463 kcal day^−1^; 95% CI: −206 to 314 kcal day^−1^; *P* = 1.00) or MD+1 (1941 ± 528 kcal day^−1^; 95% CI: −75 to 445 kcal day^−1^; *P* = 0.26). There was no significant difference in absolute EI between MD and MD+1 (95% CI: −129 to 391; *P* = 0.67) (Figure 3a). There were also no significant differences in EI between MD−1, MD or MD+1 when analysed relative to BM (35 ± 8.6 kcal kg^−1^ day^−1^, 34.2 ± 7.8 kcal kg^−1^ day^−1^ and 31.9 ± 8.3 kcal kg^−1^ day^−1^, respectively; all *P* > 0.05) (Figure 3b). Total CHO intake was significantly lower on MD+1 (213 ± 63 g day^−1^), in comparison to both MD−1 (265 ± 67 g day^−1^; 95% CI: 18–86 g day^−1^; *P* < 0.01) and MD (254 ± 67 g day^−1^; 95% CI: 7–74 g day^−1^; *P* = 0.012) (Figure 3c). Relative to BM, CHO intake was also significantly lower on MD+1 (3.5 ± 1 g kg^−1^ day^−1^), in comparison to MD−1 (4.4 ± 1.1 g kg^−1^ day^−1^; 95% CI: 0.3–1.4 g kg^−1^ day^−1^; *P* < 0.01) and MD (4.2 ± 1.1 g kg^−1^ day^−1^; 95% CI: 0.1–1.2 g kg^−1^ day^−1^; *P* = 0.01) (Figure 3d).

### 3.5 DLW derived energy intake versus RFPM

**FIGURE 4:**
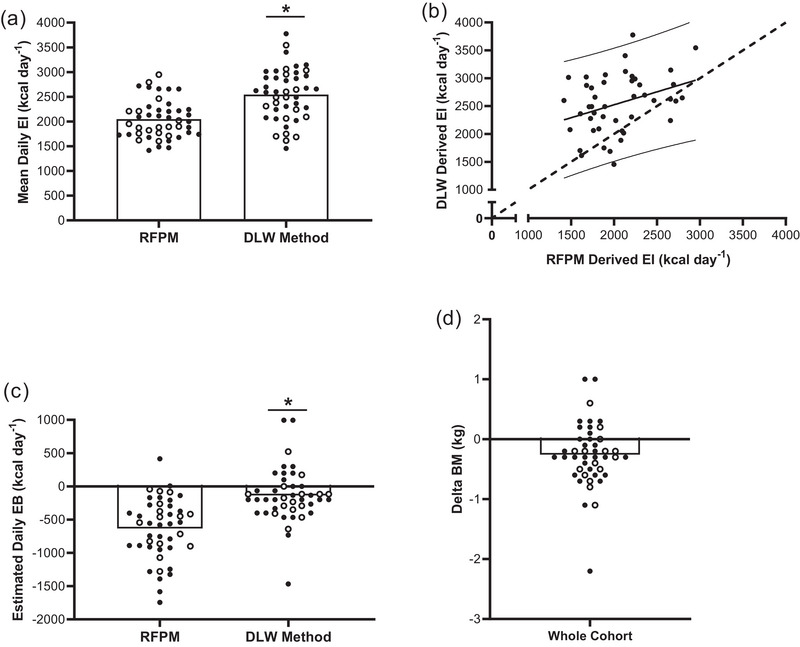
(a) Estimated daily EI using the RFPM and DLW method, (b) change in BM during the DLW derived EI assessment period, (c) estimated EB using the RFPM and DLW method, and (d) the strength of association between DLW and RFPM EI measurements (*n* = 45 for all variables). All individual circles represent an individual player, with open circles representing U17s (*n = *17) and filled circles (*n = *28) representing U19s. *Significant difference between methods (*P < *0.05).

The mean daily EI using the RFPM of 2047 ± 383 kcal day^−1^ was significantly lower than EI estimated using the DLW technique (2545 ± 518 kcal day^−1^; *P* < 0.01), representing a mean daily Δ of 499 ± 526 kcal day^−1^. This corresponds to a 25% difference between methods and a 22% error when using the RFPM, as calculated where the DLW method is assumed as the true value (Figure 4a and Table 4). There was a significant moderate positive correlation between the DLW and RFPM EI measurements (*r* = 0.34; 95% CI: 0.06–0.58; *P* = 0.02). However, the random error associated with each measurement was relatively large at 498 kcal day^−1^, with the 95% PI for the RFPM EI method being 1026 (2051) kcal day^−1^. The RFPM EI method demonstrated the presence of both unacceptable fixed (1595; 95% CI: 782–2408) and proportionate (0.46; 95% CI: 0.07–0.86) biases, highlighting under and overestimation of values versus the DLW EI method (Figure 4b). This discrepancy between methods was reflected when converted to EB, as there was no significant correlation between mean daily EB when calculated using the RFPM (−634 ± 434 kcal day^−1^), in comparison to the DLW technique (−135 ± 417 kcal day^−1^; *r* = 0.24; 95% CI: 0.06–0.50; *P* = 0.11) (Figure 4c). During the DLW derived EI assessment period there was a significant change in BM, with BM decreasing from Day 0 (61.3 ± 7.5 kg) to Day 7/8 (61.0 ± 6.5 kg; 95% CI: 0.1–0.4; *P* < 0.01) (Figure 4d).

**TABLE 4:** Individual data including BM at baseline, change in BM during the DLW derived EI assessment period, TDEE during the DLW derived EI assessment period, EI measured using the RFPM assessment period, estimated EI using the DLW method, delta difference between both EI methods, percentage difference and percentage error with the DLW method assumed to be the true value.

Player	Baseline BM (kg)	BM change (kg)	TDEE (kcal day^−1^)	EI (RFPM) (kcal day^−1^)	EI (DLW) (kcal day^−1^)	ΔEI: DLW minus RFPM (kcal day^−1^)	Difference (%)	Error (%)
Player 1	64	1.0	2781	2218	3776	1558	52.0	41.3
Player 2	54.6	1.0	2409	2130	3404	1274	46.0	37.4
Player 3	67.5	0.6	3022	2949	3544	595	18.3	16.8
Player 4	80.1	0.3	2687	2692	2887	195	7.0	6.8
Player 5	57.9	0.3	2625	1882	2924	1042	43.4	35.6
Player 6	63.6	0.3	2689	2243	2988	745	28.5	24.9
Player 7	66.5	0.2	2921	2132	3120	988	37.6	31.7
Player 8	59.3	0.2	2628	1737	2827	1089	47.7	38.5
Player 9	60.6	0.2	2136	1873	2310	437	20.9	18.9
Player 10	57.2	0.1	2922	1677	3021	1345	57.2	44.5
Player 11	74.0	0.0	3035	2210	3035	825	31.4	27.2
Player 12	62.0	0.0	2880	2299	2880	581	22.4	20.2
Player 13	54.0	0.0	2242	2657	2242	−415	17.0	18.5
Player 14	67.3	−0.1	3214	2660	3147	487	16.8	15.5
Player 15	51.1	−0.1	2373	2203	2306	103	4.6	4.5
Player 16	56.9	−0.2	2497	1608	2363	756	38.1	32.0
Player 17	68.2	−0.2	3177	1898	3061	1163	46.9	38.0
Player 18	58.6	−0.2	2609	1892	2492	600	27.4	24.1
Player 19	51.0	−0.2	2178	1760	2062	302	15.8	14.6
Player 20	60.7	−0.2	3070	2208	2953	745	28.9	25.2
Player 21	49.2	−0.2	2226	1823	2093	270	13.8	12.9
Player 22	55.9	−0.3	1925	1882	1750	−132	7.3	7.5
Player 23	78.5	−0.3	2691	2356	2697	341	13.5	12.6
Player 24	66.1	−0.3	2825	1470	3014	1544	68.9	51.2
Player 25	64.3	−0.3	2897	1739	2491	752	35.6	30.2
Player 26	63.4	−0.3	3214	2657	2625	−32	1.2	1.2
Player 27	55.1	−0.3	2442	2020	2242	222	10.4	9.9
Player 28	58.6	−0.3	3070	1679	2870	1191	52.4	41.5
Player 29	52.1	−0.3	2479	1726	2279	553	27.6	24.3
Player 30	58.5	−0.4	2880	2793	2647	−146	5.4	5.5
Player 31	56.5	−0.4	2858	2719	2591	−128	4.8	4.9
Player 32	57.5	−0.5	2782	1710	2490	780	37.1	31.3
Player 33	57.8	−0.5	2669	1768	2377	610	29.4	25.6
Player 34	64.5	−0.5	2387	2093	2054	−39	1.9	1.9
Player 35	43.3	−0.6	2052	1603	1702	99	6.0	5.8
Player 36	70.6	−0.6	3059	1778	2659	881	39.7	33.1
Player 37	68.6	−0.6	2287	2080	1887	−193	9.7	10.2
Player 38	63.0	−0.6	3001	1416	2601	1185	59.0	45.6
Player 39	70.0	−0.7	3009	2464	2601	137	5.4	5.3
Player 40	67.7	−0.7	3140	2230	2674	443	18.1	16.6
Player 41	62.0	−0.7	2485	2112	2019	−93	4.5	4.6
Player 42	55.0	−0.8	2081	1622	1614	−8	0.5	0.5
Player 43	56.3	−1.1	2811	1487	2077	591	33.1	28.4
Player 44	59.1	−1.1	2329	1953	1687	−266	14.6	15.8
Player 45	69.3	−2.2	2923	1999	1456	−543	31.4	37.3
**Mean ± SD**	**61.3 ± 7.4**	**−0.3 ± 0.5**	**2680 ± 343**	**2047 ± 383**	**2545 ± 518**	**499 ± 528**	**25 ± 18**	**22 ± 14**

### 3.6 Estimated energy availability

Mean daily EA was significantly higher (*P* < 0.01) when using EI data derived from the DLW technique (48 ± 14 kcal kg^−1^ FFM day^−1^), in comparison to using EI data derived from the RFPM (37 ± 8 kcal kg^−1^ FFM day^−1^). When using the RFPM, there was a prevalence of 7 (18%), 27 (68%) and 6 (15%) players in a state of optimal EA, reduced EA and LEA, respectively. In contrast, when the DLW method was employed, 23 (58%) players were in a state of optimal EA, 15 (38%) reduced EA and only 2 (5%) in a state of LEA.

## 4 DISCUSSION

In using the DLW method, the aim of the present study was to quantify TDEE, EI and EA of female adolescent soccer players. Confirming our hypothesis, these data demonstrate that DLW‐derived estimates of EI were significantly greater than estimates derived from the RFPM. Accordingly, the prevalence of players categorised with LEA (albeit as classified from historical laboratory‐based values) was significantly reduced when using DLW‐derived estimates of EI (58% optimal, 38% reduced and 5% low) compared with participants’ self‐reporting dietary intake (18% optimal, 68% reduced and 15% low). From a practical perspective, the present data not only extend our understanding of the daily energy requirements of female soccer players training and competing at an international standard, but also provide greater methodological rigour to further evaluate the prevalence of LEA in this population of athletes. In that sense, our data offer caution to both researchers and practitioners alike, supporting recent recommendations that LEA should not be used directly as a REDs diagnostic tool (Ackerman et al., 2023).

To address our aim, we sampled the largest cohort of female players studied to date (*n* = 45), as assessed in a ‘training camp’ environment during which they also played competitive games representing their national team. In relation to TDEE, we report a mean absolute and relative expenditure of 2683 ± 324 kcal day^−1^ and 45 ± 4 kcal kg^−1^ day^−1^, respectively (Figure 1a,b). The present data compare favourably with our previous observations from adult players (from the same national team) where absolute and relative TDEE was 2693 ± 432 kcal day^−1^ and 43 ± 6 kcal kg^−1^ day^−1^, respectively, as reported over a similar time course of data collection (Morehen et al., 2021). Such similarity between age groups is not surprising when considering the comparable values for total BM and FFM of the players studied here (60.8 ± 7 and 45.0 ± 5.1 kg) and those from our previously studied adult cohort (62.1 ± 4.7 and 43.2 ± 3.4 kg). In this regard, it is noteworthy that the TDEE reported here was also positively correlated with players’ BM, FFM and stature, as opposed to crude markers of training volume, such as total distance or training duration (Figure 2).

In using the RFPM to examine players’ self‐reported EI during a 3‐day assessment period, we report a mean absolute EI of 2047 ± 511 kcal day^−1^ (range: 1456–3776 kcal). Such data also compare favourably with our previous assessments of both adult (Morehen et al., 2021) and adolescent (McHaffie et al., 2023) players, where mean absolute EI was 1923 ± 232 and 2053 ± 486 kcal day^−1^ during a 4‐day and 10‐day assessment period, respectively. However, when the DLW method (and changes in BM over the initial 7–8 days of training) was used to derive estimates of EI, we observed considerable discrepancy between methods (Figure 4a,b). When examined at group level, our data demonstrate a mean difference of 499 + 526 kcal day^−1^ between methods, thus representing a percentage difference and percentage error of 25% and 22%, respectively. The error reported here also compares well with that identified in recent studies conducted on Dutch (Brinkmans et al., 2024) and Norwegian (Dasa et al., 2023) female soccer players where an error of 22% and 20% was reported, respectively. Whilst we acknowledge the limitation of obtaining dietary records over a 3‐day period (as opposed to the similar time course where DLW and BM was used), our data provide further evidence in support of the inaccuracies of utilising the RFPM to make inferences on absolute EI within real world environments. This is especially apparent when considering the large range of variance between methods when examined at an individual participant level (Table 4). Although the potential sources of error in both retrospective and prospective assessment methods have been frequently documented (Capling et al., 2017; Gemming et al., 2014; Livingstone & Black, 2003; Martin et al., 2012; Poslusna et al., 2009; Rollo et al., 2016; Thompson et al., 2010), the use of the RFPM has become particularly popularised within the field of sport nutrition research and applied practice. In addition to errors associated with both participant (e.g., reporting of food ‘leftovers’, failure to report snacks and drinks, etc.) and researcher burden (e.g., provision of frequent daily prompts), it is noteworthy that a large proportion of error is also likely attributable to the ability of the coder to estimate portion sizes and/or ‘hidden’ ingredients such as oil used within the cooking process. Indeed, we previously reported that applied sport nutrition practitioners (*n* = 48) can under‐estimate the energy content of meals by approximately 10%, with individual variation between coders ranging from −47% to +18% (Stables et al., 2021). Such data collectively demonstrate the possibility of both intentional and unintentional under‐reporting by participants, but also considers the methodological challenges inherent in accurately measuring dietary intake. Our data also reflect recent research over a longer period of time, that also identified interindividual variance and considerable differences between direct and indirect assessment of energy balance (Müller et al., 2023).

**FIGURE 5:**
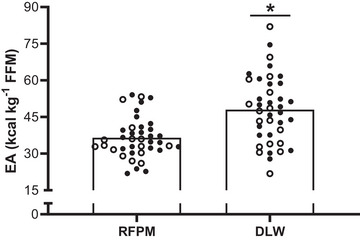
Mean daily EA calculated using the RFPM and DLW method for all outfield players (*n* = 40). All individual circles represent an individual player, with open circles representing U17s (*n = *15) and filled circles representing U19s (*n = *25). *Significant difference between methods (*P <* 0.05).

In accordance with the reported differences in EI between methods, it follows that the pattern of EA reported within the present cohort of players is also dependent on the methodological approach (Figure 5). Indeed, the prevalence of players categorised with low, reduced and optimal EA (albeit as classified from historical laboratory‐based values of <30, 30–45 and >45 kcal kg^−1^ FFM day^−1^, respectively) was significantly different when using DLW derived estimates of EI (58%, 38% and 5%, respectively), compared with participants’ self‐reporting dietary intake (18%, 68% and 15%, respectively), notably resulting in lower prevalence of LEA. When considering the present data in the context of previous reports of LEA (as estimated using self‐reported methods) from elite female soccer players representative of both international standard adult (88%) and adolescent players (34%), as well as within domestic level competition in Norway (23% and 36% on training and match days, respectively) (Dasa et al., 2023) and England (23%) (Moss et al., 2021), it is suggested that the prevalence of LEA within female soccer players and athletes as a whole may have been overestimated within the literature. In this regard, the present data should therefore serve as a caution to both researchers and practitioners alike, given the potential to underestimate EA and inflate the incidence of LEA if a given EA threshold is used, especially in those instances where they may present with symptoms outlined within the conceptual health and performance REDs models that may not be attributable to LEA in the first instance (Mountjoy et al., 2023a; Parker et al., 2022). Our data also support the notion that direct assessment of EI should not be used within free living situations to calculate EA, as recently highlighted by Brinkmans et al. (2024).

Additionally, it should also be noted that this field is further complicated in that the categorisation of LEA (as <30 kcal kg^−1^ FFM day^−1^) is typically based on laboratory studies with homogeneous patterns of EI and exercise‐related expenditure (Burke et al., 2018). In contrast, athletes living under free‐living conditions (such as those studied here) typically present daily variations in training volume and intensity (and hence EEE), albeit we acknowledge that athletes may not always adjust their EI in accordance with fluctuations in training load (McHaffie et al., 2023). Therefore, the findings of this study further the argument against using universal categories to define LEA (i.e., <30 kcal kg^−1^ FFM day^−1^) (De Souza et al., 2022; Lieberman et al., 2018; Salamunes et al., 2024), given the discrepancy in values, depending on the method used to measure EI. In addition to EI, the methodology used to measure EEE and FFM may also result in variable outcomes (Ackerman et al., 2023), and challenges exist surrounding the definition of ‘exercise’ (Areta et al., 2021). Although the present study is advantageous in that we studied an elite athlete cohort in free living conditions, it is noteworthy that measurement error associated with RMR (i.e., prediction equations were used as opposed to true measurement), EEE (a correction factor was employed) and body mass (we acknowledge that daily hydration status was not measured to verify that changes were not due to small changes in total body water content) can all collectively influence estimated PAL and EA values. As such, we also present supplementary data in Supporting information Tables S1 and S2 to demonstrate how a change in RMR (±10%) and body mass (±1%; this value was chosen as all players did not show any day‐to‐day variation greater than 1%) can affect estimates of PAL and EA. When taken together, it is apparent that there is a definitive need for further research in multiple athletic cohorts, using longitudinal research designs, and with valid assessments of RMR, EEE, total body water content and EI, in order to allow for a more rigorous evaluation of the prevalence of LEA within both female and male athletes.

From a practical perspective, the assessment of TDEE also provides further evidence to formulate population and sport‐specific nutritional guidelines. Indeed, on the basis of a recommended daily protein intake of 1.6 g kg^−1^ (Morton et al., 2018) and daily fat intake equivalent to 30% of EI (Collins et al., 2021), it can be estimated that daily CHO intakes for the players studied here are likely in the region of 5 g kg^−1^. That said, although such daily CHO intakes are likely sufficient to support daily training requirements, it is also suggested that CHO intake be increased to at least 6–8 g kg^−1^ on the day before match play, on match day itself and on the day after match play, so as to promote sufficient muscle glycogen storage for performance and recovery. However, consistent with previous assessments of self‐reported dietary intake (also using the RFPM) in both adolescent (McHaffie et al., 2023) and adult (Morehen et al., 2021) international soccer players, as well as elite players at club level (Brinkmans et al., 2024; Dasa et al., 2023; Moss et al., 2021), we also acknowledge that players’ habitual CHO intakes are not sufficient to promote match play performance and recovery. Indeed, only one participant studied here self‐reported a mean daily CHO intake >6 g kg^−1^ during the 3‐day period surrounding match play. Notwithstanding the error associated with self‐reporting estimates of energy and CHO intake (as previously discussed), our data are still in support of the assertion that female players are likely ‘under‐fuelling for the work required’ (i.e., match play) (McHaffie et al., 2023), the reasons for which may be due to a lack of awareness of nutritional guidelines and/or deliberate and intentional behaviours based on CHO fear, body image challenges and misconceptions surrounding body composition (McHaffie et al., 2022). In this regard, our data provide further rationale for the formulation of player and stakeholder specific education and behaviour change interventions that aim to promote a positive nutrition culture within the women's game.

In summary, the present data provide the first report to assess TDEE of female adolescent soccer players competing at an elite international standard and is the largest DLW study conducted in female athletes to date. Importantly, the use of the DLW method also allowed for a comparison between DLW derived estimates of EI and those estimates of EI derived from the RFPM. We conclude that the use of the latter method significantly underestimates EA within this population particularly when universal thresholds are applied.

## AUTHOR CONTRIBUTIONS

Conceptualisation and design of the study was conducted by Samuel J. McHaffie, James P. Morton, Christopher Rosimus, Martin Evans and Carl Langan‐Evans. Acquisition of data was conducted by Samuel J. McHaffie, Ruth Waghorn, Martin Evans, Matthew Cuthbert and James Grant. Analysis of data was conducted by Samuel J. McHaffie, Carl Langan‐Evans, Catherine Hambly and John R. Speakman. Samuel J. McHaffie wrote the first draft of the manuscript, which was revised with Carl Langan‐Evans, José L. Areta, Juliette A. Strauss and James P. Morton. The final draft of the manuscript was revised by all authors, who have read and agreed to the published version of the manuscript and agree to be accountable for all aspects of the work in ensuring that questions related to the accuracy or integrity of any part of the work are appropriately investigated and resolved. All persons designated as authors qualify for authorship, and all those who qualify for authorship are listed.

## CONFLICT OF INTEREST

J.P.M. is a consultant to Science in Sport (plc).

## Supporting information

Tables S1 and S2.

## Data Availability

The data that support the findings of this study are available from the corresponding author upon reasonable request.
