# Supplementary material for: Energy expenditure, intake and availability in female soccer players via doubly labelled water: Are we misrepresenting low energy availability?
Source: Exp Physiol. 2024 Aug 15;110(11):1705–20. doi: 10.1113/EP091589 (PMC12576014; doi:10.1113/EP091589)
Supplement: Supplementary file 1 — Tables S1 and S2. [file EPH-110-1705-s001.pdf]

Table S1. Individual data ranges, including potential RMR equation error (±10%) and the subsequent impact on PAL, mean daily EEE (both including and excluding the Dasa et al. 2022 correction factor) and EA range using both EI assessment methods.

RMR range based on RMR equation error (kcal): This column represents the range of RMR values considering a potential ±10% error margin in the RMR equation.

PAL range based on RMR equation error: This column represents the range in PAL values considering the potential error in the RMR equation.

Mean daily EEE range based on RMR equation error (kcal.day^{-1}): This column represents the range in mean daily EEE considering potential error in the RMR equation.

Mean daily EEE range based on RMR equation error, excluding correction factor (kcal.day^{-1}): This column represents the range of mean daily EEE values, excluding the correction factor, considering the potential error in the RMR equation.

EA range based on RMR equation error, using DLW-derived EI (kcal.kg^{-1} FFM.day^{-1}): This column provides the range of EA values based on the potential error in the RMR equation, using DLW derived EI.

EA range based on RMR equation error, using RFPM-derived EI (kcal.kg^{-1} FFM.day^{-1}): This column represents the range of EA values based on the potential error in the RMR equation, using EI derived from the RFPM.

Player	RMR range based on RMR equation error (kcal)	PAL range based on RMR equation error	Mean daily EEE range based on RMR equation error (kcal·day^{-1})	Mean daily EEE range based on RMR equation error, excluding correction factor (kcal·day^{-1})	EA range based on RMR equation error, using DLW derived EI (kcal·kg^{-1} FFM·day^{-1})	EA range based on RMR equation error, using RFPM derived EI (kcal·kg^{-1} FFM·day^{-1})
Player 1	1362-1665	1.8-2.2	492-480	439-427	31-32	34-35
Player 2	1243-1519	1.7-2.1	277-265	245-234	74-76	50-51
Player 3	1389-1697	1.8-2.2	426-413	379-366	37-38	52-53
Player 4	1471-1797	1.5-1.9	N/A (GK)	N/A (GK)	N/A (GK)	N/A (GK)
Player 5	1265-1546	1.6-2.0	432-420	385-373	52-54	37-38
Player 6	1325-1619	1.7-2.1	437-425	390-378	46-47	38-39
Player 7	1365-1668	1.8-2.2	256-244	226-214	29-30	40-41
Player 8	1278-1562	1.8-2.2	265-253	234-222	64-65	36-37
Player 9	1291-1578	1.5-1.8	256-244	226-214	42-43	32-33
Player 10	1287-1573	1.9-2.3	517-505	462-450	44-46	28-29
Player 11	1419-1734	1.8-2.2	382-369	340-327	46-47	32-33
Player 12	1309-1600	1.8-2.2	389-377	346-334	29-31	41-42
Player 13	1244-1521	1.4-1.7	569-557	509-497	60-62	53-55
Player 14	1373-1679	1.8-2.2	N/A (GK)	N/A (GK)	N/A (GK)	N/A (GK)
Player 15	1244-1520	1.8-2.2	244-233	216-204	40-41	54-55
Player 16	1254-1533	1.7-2.0	;189-178	166-154	44-45	33-34
Player 17	1360-1662	1.9-2.4	368-356	327-315	31-32	29-30
Player 18	1291-1578	1.7-2.0	364-352	324-312	34-35	34-35
Player 19	1218-1489	1.5-1.8	267-256	237-225	47-48	39-40
Player 20	1328-1623	1.7-2.1	394-381	350-338	65-66	39-40
Player 21	1203-1471	1.3-1.6	357-346	318-307	81-82	37-39
Player 22	1255-1533	1.4-1.7	476-464	425-413	57-59	32-33
Player 23	1484-1813	1.6-2.0	N/A (GK)	N/A (GK)	N/A (GK)	N/A (GK)
Player 24	1373-1678	1.6-2.0	N/A (GK)	N/A (GK)	N/A (GK)	N/A (GK)
Player 25	1347-1647	1.7-2.0	345-333	307-294	32-33	42-43
Player 26	1342-1640	1.8-2.2	187-174	163-151	51-52	55-56
Player 27	1272-1555	1.4-1.7	301-290	267-255	48-49	41-42
Player 28	1293-1580	1.8-2.2	378-366	336-324	56-57	32-33
Player 29	1210-1479	1.7-2.0	418-406	372-361	58-59	35-36
Player 30	1298-1586	1.8-2.2	508-496	453-441	42-43	54-55
Player 31	1249-1527	1.8-2.2	505-494	451-440	54-55	55-56
Player 32	1269-1551	1.7-2.1	313-301	277-266	51-52	31-33
Player 33	1277-1560	1.7-2.1	491-479	439-427	54-55	29-31
Player 34	1350-1650	1.5-1.8	N/A (GK)	N/A (GK)	N/A (GK)	N/A (GK)
Player 35	1152-1408	1.5-1.8	529-518	473-462	56-58	29-30
Player 36	1406-1718	1.8-2.2	373-360	331-318	36-37	27-28
Player 37	1371-1675	1.4-1.7	262-249	231-218	48-48	39-40
Player 38	1317-1610	1.7-2.1	386-373	343-331	75-76	23-24
Player 39	1419-1734	1.9-2.3	382-369	340-326	38-39	41-42
Player 40	1370-1675	1.8-2.2	313-300	277-264	49-50	37-38
Player 41	1297-1585	1.6-2.0	345-333	306-294	57-58	29-30
Player 42	1197-1463	1.5-1.9	247-235	218-207	28-29	32-33
Player 43	1267-1548	1.7-2.0	232-220	205-193	58-59	27-28
Player 44	1294-1581	1.8-2.2	369-357	328-316	53-55	37-38
Player 45	1371-1676	1.6-2.0	307-294	272-259	62-63	38-39
**Mean ± SD**	**1311±71 - 1602±88**	**1.7 ± 0.2 - 2.0 ± 0.2**	**352 ± 97 - 364 ± 97**	**269 ± 146 - 281 ± 146**	**49 ± 13 - 50 ± 13**	**38 ± 9 - 39 ± 9**

Table S2. Individual data range including potential BM fluctuations (±1%), as a result of changes in hydration status and the subsequent impact on DLW derived EI and EA. The mean and SD of daily fluctuation percentages are also reported for each participant to demonstrate that ±1% is a representative error range within the context of this study. For context, no player exceeded ±1% on any given day.

DLW-derived EI (kcal.day^{-1}): Represents daily EI derived using the DLW method.

Mean daily BM fluctuation percentage: Represents the mean daily percentage change in BM, as calculated from consecutive days of measurement.

DLW-derived EI range based on ±1% BM (kcal.day^{-1}): Represents the potential range of DLW-derived EI, accounting for a ±1% daily fluctuation in BM due to changes in hydration status (the ±1% was applied to the measured BM on the final day of data collection that was used for assessment of DLW derived EI).

EA range based on ±1% BM (kcal.kg^{-1} FFM.day^{-1}): Provides the potential range of EA, considering the ±1% fluctuation in BM.

Player	DLW derived EI (kcal·day^{-1})	Mean and SD daily BM fluctuation percentage	DLW derived EI range based on ±1% BM (kcal·day^{-1})	EA range based on ±1% BM (kcal.kg^{-1} FFM·day^{-1})
Player 1	3776	0.4 ± 0.4	3129-4423	60-89
Player 2	3404	0.4 ± 0.3	2851-3958	50-74
Player 3	3544	0.2 ± 0.6	2951-4137	67-97
Player 4	2887	0.1 ± 0.2	2351-3423	52-80
Player 5	2924	0.1 ± 0.2	2345-3503	N/A (GK)
Player 6	2988	0.1 ± 0.2	2352-3624	36-58
Player 7	3120	0.1 ± 0.6	2456-3784	45-72
Player 8	2827	0.1 ± 0.5	2235-3419	38-65
Player 9	2310	0.1 ± 0.3	1781-2840	27-52
Player 10	3021	0.0 ± 0.1	2451-3592	46-72
Player 11	3035	0.0 ± 0.3	2390-3679	52-87
Player 12	2880	0.0 ± 0.5	2281-3479	39-65
Player 13	2242	0.0 ± 0.1	1882-2602	30-45
Player 14	3147	0.0 ± 0.4	2699-3595	31-44
Player 15	2306	-0.1 ± 0.1	1966-2646	N/A (GK)
Player 16	2363	-0.1 ± 0.3	1985-2741	35-50
Player 17	3061	-0.1 ± 0.3	2664-3457	52-70
Player 18	2492	0.0 ± 0.3	2152-2833	36-51
Player 19	2062	-0.1 ± 0.6	1765-2358	28-40
Player 20	2953	0.0 ± 0.6	2600-3306	53-71
Player 21	2093	-0.1 ± 0.2	1766-2420	36-51
Player 22	1750	-0.1 ± 0.5	1426-2075	24-38
Player 23	2697	0.0 ± 0.6	2176-3218	40-59
Player 24	3014	0.0 ± 0.2	2575-3453	N/A (GK)
Player 25	2491	0.1 ± 0.6	2065-2918	N/A (GK)
Player 26	2625	-0.1 ± 0.3	2204-3046	49-72
Player 27	2242	-0.1 ± 0.5	1876-2607	26-40
Player 28	2870	-0.1 ± 0.4	2481-3259	43-58
Player 29	2279	-0.1 ± 0.6	1934-2624	33-49
Player 30	2647	0.1 ± 0.8	2308-2985	47-63
Player 31	2591	-0.1 ± 1.0	2217-2965	37-54
Player 32	2490	-0.1 ± 0.6	2158-2823	42-59
Player 33	2377	-0.1 ± 0.4	2043-2711	41-57
Player 34	2054	-0.1 ± 0.4	1627-2480	27-41
Player 35	1702	-0.2 ± 0.6	1453-1951	N/A (GK)
Player 36	2659	-0.2 ± 0.0	2192-3126	42-63
Player 37	1887	-0.2 ± 0.7	1434-2341	20-40
Player 38	2601	-0.2 ± 0.4	2185-3017	52-74
Player 39	2601	-0.1 ± 0.5	2197-3005	39-56
Player 40	2674	-0.2 ± 0.5	2227-3120	39-61
Player 41	2019	-0.2 ± 0.3	1610-2427	24-39
Player 42	1614	-0.2 ± 0.3	1298-1931	15-29
Player 43	2077	-0.4 ± 0.4	1709-2445	34-54
Player 44	1687	-0.3 ± 0.3	1349-2025	23-39
Player 45	1456	-0.3 ± 0.4	1009-1904	17-38
**Mean ± SD**	**2545 ± 518**	**0 ± 0.2**	**2106 ± 451 - 2984 ± 597**	**38 ± 12 - 58 ± 16**
